# Well‐Differentiated, Low‐Grade Subcutaneous Liposarcoma With a Fatal Outcome in a Dog

**DOI:** 10.1002/vms3.70939

**Published:** 2026-04-07

**Authors:** Peres Ramos Badial, Thiago André Salvitti De Sá Rocha, Bárbara Ataíde Adorno, Hugo Henrique Ferreira, Didier Quevedo Cagnini

**Affiliations:** ^1^ Department of Pathobiology and Population Medicine College of Veterinary Medicine, Mississippi State University Starkville Mississippi USA; ^2^ Department of Veterinary Medicine Federal University of Jataí (UFJ) Jataí Goiás Brazil; ^3^ Lurion Lab ‐ Anatomopatologia Veterinaria Jataí Goiás Brazil; ^4^ Department of Veterinary Clinics School of Veterinary Medicine and Animal Science, São Paulo State University (UNESP) Botucatu São Paulo Brazil

**Keywords:** dogs, liposarcoma, soft tissue neoplasms, subcutaneous tissue

## Abstract

A 10‐year‐old intact male Rottweiler was presented with a large, rapidly growing subcutaneous mass on the right thoracic limb. Physical examination revealed a firm, raised, non‐ulcerated mass extending from the proximal scapula to the distal humerus. Cytological evaluation identified a highly cellular population of vacuolated cells with marked anisocytosis and anisokaryosis, consistent with a lipid‐rich mesenchymal neoplasm. Histopathology of incisional biopsies confirmed a well‐differentiated, low‐grade subcutaneous liposarcoma. The owner declined therapeutic and surgical treatments. The dog died 3 months after diagnosis, with clinical signs suggestive of metastatic disease. This case highlights the importance of comprehensive tumour assessment for accurate grading, staging and effective management of canine soft tissue sarcomas, including liposarcomas. Liposarcoma should be considered a differential diagnosis for subcutaneous masses in the proximal limbs of dogs. Early diagnosis, accurate grading, complete staging and prompt surgical intervention, including limb amputation if warranted, are critical even for histologically well‐differentiated, low‐grade variants.

## Graphical abstract

This case report describes a rapidly growing subcutaneous liposarcoma with a fatal outcome in a 10‐year‐old intact male Rottweiler. The neoplasm was suspected cytologically and confirmed histologically. Despite its well‐differentiated, low‐grade features, the dog died within three months of diagnosis, highlighting the potential for metastasis of such tumours. Early diagnosis, accurate grading, complete staging and prompt surgical intervention, including limb amputation when indicated, are essential even for low‐grade liposarcomas.

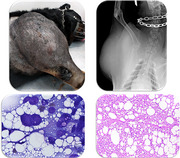

## 1 Introduction

Liposarcomas are soft tissue sarcomas characterized by adipocytic differentiation arising from lipoblasts or lipocytes (Baez et al. 2004; Dennis et al. 2011; Hendrick 2016). These neoplasms are infrequently reported in dogs and most commonly occur in the subcutis of the axial and proximal appendicular skeleton (Baez et al. 2004; Hendrick 2016). Complete surgical excision is typically curative, recurrence is uncommon following surgery and metastases are generally considered rare (Baez et al. 2004; Dennis et al. 2011; Hendrick 2016). However, some liposarcoma subtypes in dogs are locally invasive and may have an increased potential for recurrence and metastatic disease (Baez et al. 2004; Dennis et al. 2011; Hendrick 2016).

Canine liposarcomas are typically classified into well‐differentiated, myxoid and pleomorphic subtypes, based on degree of adipocytic differentiation, cellular morphology and degree of atypia (Hendrick 2016). Recent studies have also described canine liposarcomas with histologic features consistent with a dedifferentiated morphology (Avallone et al. 2016; Muscatello et al. 2024), as described in human pathology (Weiss and Goldblum 2008). Well‐differentiated liposarcomas most closely resemble normal adipose tissue and are generally considered to exhibit the most indolent behaviour, whereas the remaining subtypes are thought to have a more aggressive biological potential and a shorter median survival time (Baez et al. 2004; Avallone et al. 2016). While the histologic subtype in human liposarcomas reliably predicts the biological behaviour and overall 5‐year survival rate (Coindre et al. 2001; Weiss and Goldblum 2008), no definitive evidence supports a similar association in dogs (Baez et al. 2004). Although the prognostic relevance of histologic subtype remains unproven, survival time is associated with the completeness of surgical margins (Baez et al. 2004; Hendrick 2016).

This report documents a case of a well‐differentiated, low‐grade liposarcoma in the proximal thoracic limb of a dog, with clinical progression to presumed metastatic disease in the absence of treatment and spontaneous death after 3 months of diagnosis. Although histologically low‐grade, the tumour exhibited unexpectedly aggressive behaviour, highlighting the limitations of incisional biopsy in isolation for accurate grading and prognostication. The discrepancy between the biopsy findings and the clinical outcome underscores the importance of accurate grading and staging, as well as prompt treatment interventions.

## 2 Case Presentation

A 10‐year‐old, 46.5 kg, intact male Rottweiler was presented for evaluation of a mass on the right thoracic limb. The owner had first noticed the mass three months prior, and it had enlarged substantially over the past month. According to the owner, the dog remained non‐lame and appeared healthy.

**FIGURE 1:**
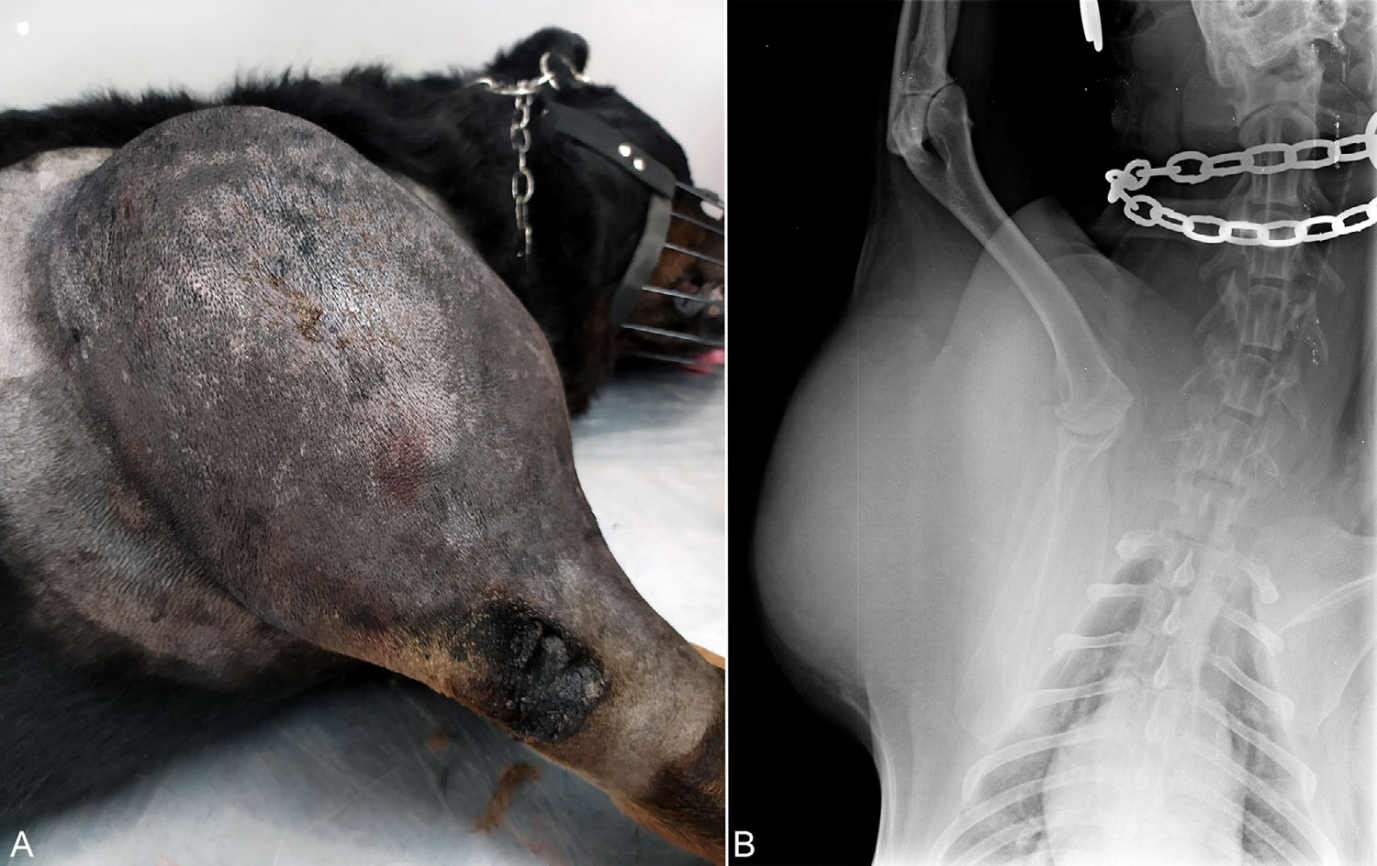
A 10‐year‐old intact male Rottweiler with a large, cutaneous, mildly painful liposarcoma extending from the proximal third of the right scapula to the distal third of the right humerus. (A) The liposarcoma is approximately 23 cm × 19 cm × 12 cm, firm, raised, adhered and non‐ulcerated with a soft and slightly depressed centre. (B) Ventrodorsal radiographic projections of the right proximal thoracic limb revealing a radiopaque, mildly heterogeneous soft tissue mass adjacent to the scapular and humeral regions with evidence of periosteal reaction in the proximal humeral metaphysis.

Physical examination was not possible at the initial visit due to the dog's aggressive behaviour associated with pain in the right thoracic limb, which was managed with gabapentin (10 mg/kg PO, q 12 h for 30 days). Following the first week of therapy, the patient was bright, alert and responsive on physical examination. The dog had a large, subcutaneous, mildly painful mass in the lateral region of the right shoulder and brachium (Figure 1). The mass did not limit the limb's movement or cause lameness on gait evaluation. The remainder of the physical examination was unremarkable.

A complete blood count (CBC) revealed mild non‐regenerative anaemia and moderate lymphopenia (Table 1). The remaining CBC and serum biochemistry values were considered within normal limits (Table 1). Radiography of the right forelimb revealed a radiopaque, mildly heterogeneous soft tissue mass adjacent to the scapular and humeral regions (Figure 1). The lateral and medial borders of the proximal humeral metaphysis exhibited evidence of periosteal reaction. Thoracic radiography and abdominal ultrasonography were unremarkable.

**TABLE 1:** Complete blood count and serum chemistry at presentation and after 60 days in a 10‐year‐old intact male Rottweiler with a right thoracic limb cutaneous liposarcoma.

Complete blood count			
Parameter	First exam	Second exam	Reference range
RBC	5.49 × 10^6^/µL	2.8 × 10^6^/µL	5.7–8.4 × 10^6^/µL
Haemoglobin	10.4 g/dL	6.1 g/dL	14–18 g/dL
Haematocrit	33.0%	19%	38%–47%
MCV	60.1 fL	68 fL	60–77 fL
MCH	18.9 pg	22 pg	19–23 pg
MCHC	31.5 g/dL	32 g/dL	32–36 g/dL
Notes	None	Presence of reticulocytes	
Total WBC	8760/µL	18,590/µL	8000–16,000/µL
Segmented neutrophils	5519/µL	15,058/µL	3.680–10.880/µL
Band neutrophils	263/µL	0/µL	0–160/µL
Eosinophils	1139/µL	186/µL	60–1440/µL
Basophils	0	0	Rare
Lymphocytes	1139/µL	2231/µL	2400–7680/µL
Monocytes	701/µL	1115/µL	80–1600/µL
Platelets	328 × 10^3^/µL	79 × 10^3^/µL	200–500 × 10^3^/µL
Plasma protein	7.0 g/dL	7.2 g/dL	6.0–8.0 g/dL

Serum biochemistry			
Parameter	First exam	Second exam	Reference range
ALT	82 U/L	3060 U/L	10–88 U/L
ALP	104 U/L	199.5 U/L	10–92 U/L
Urea	35 mg/dL	152 mg/dL	15–40 mg/dL
Creatinine	0.6 mg/dL	3.8 mg/dL	0.5–1.5 mg/dL

Upon further examination, the mass in the right thoracic limb was approximately 23 cm × 19 cm × 12 cm, firm, raised, adhered and non‐ulcerated with a soft and slightly depressed centre. The mass extended from the proximal third of the right scapula to the distal third of the right humerus (Figure 1). The primary differential diagnoses for this mass were a soft tissue sarcoma or, less likely, an inflammatory or infectious process.

**FIGURE 2:**
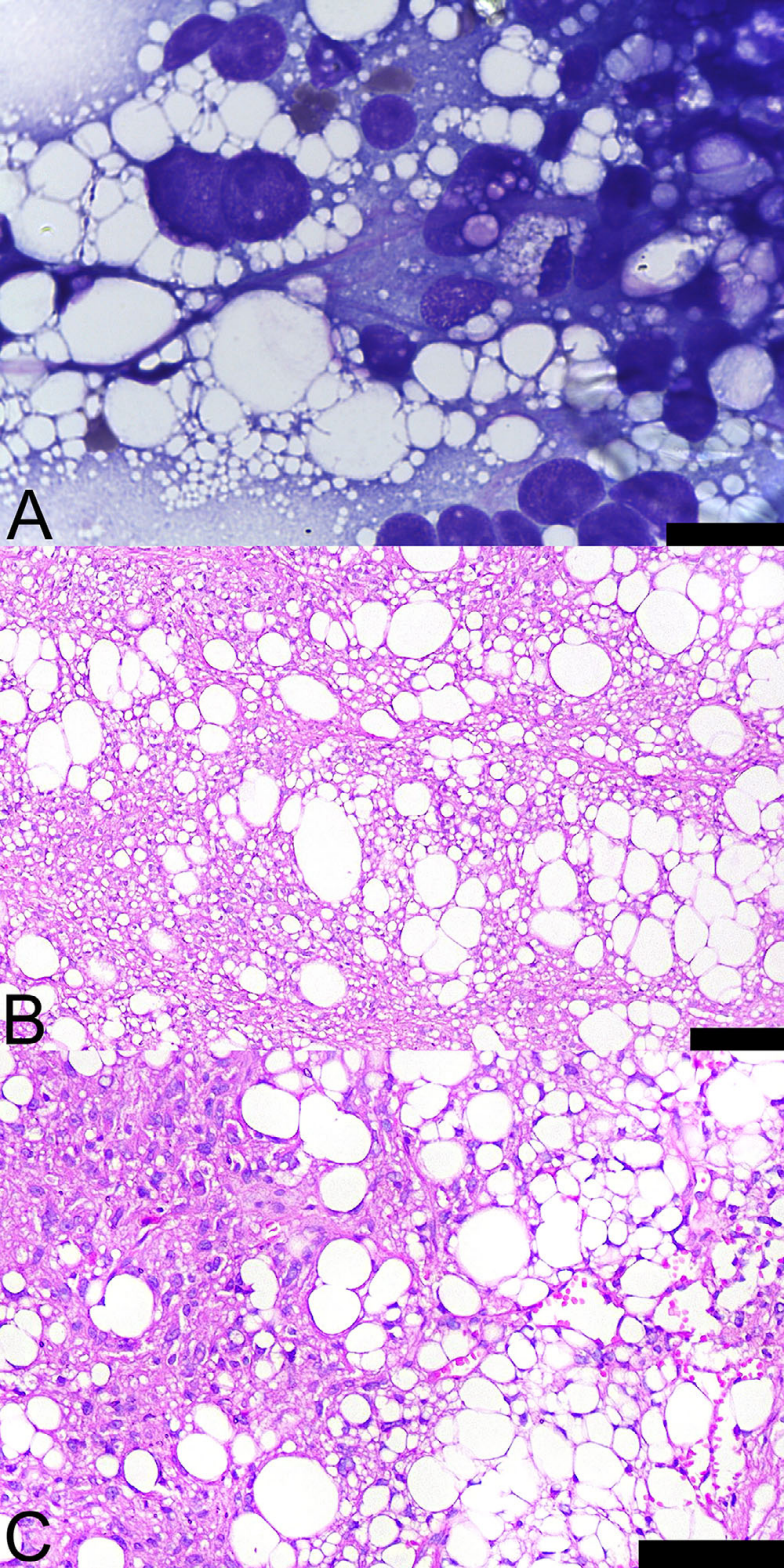
Cytopathological and histopathological evaluations of the cutaneous liposarcoma from the dog depicted in Figure 1. (A) The fine‐needle aspirate is composed of a highly cellular population of polygonal, vacuolated, moderately to markedly pleomorphic neoplastic cells often arranged in loose aggregates. Diff‐Quick stain; bar = 20 µm. (B) The dermis is effaced by a densely cellular, poorly demarcated and infiltrative population of polygonal to spindle‐shaped, markedly pleomorphic neoplastic cells arranged in solid aggregates or interlacing streams separated by a delicate fibrovascular stroma. The neoplastic cells exhibit either multiple, small, round to oval, variably discrete and clear intracytoplasmic vacuoles or a single, large, oval, discrete, clear intracytoplasmic vacuole. Nuclei are often compressed toward the periphery by these vacuoles. H&E stain; bar = 100 µm. (C) Higher magnification of the neoplastic cells illustrating the cellular features described in Figure 2B. H&E stain; bar = 100 µm.

Cytological evaluation of the mass revealed a highly cellular population of vacuolated cells, on a lipid‐rich background, often arranged in loose aggregates and rarely as individual cells (Figure 2). The cells were polygonal to oval, with indistinct borders, basophilic cytoplasm and multiple variably sized, round to oval, clear intracytoplasmic vacuoles. Nuclei were round to oval to moderately elongated and exhibited coarse chromatin with one or more prominent nucleoli. Anisocytosis was moderate, anisokaryosis was marked, and the nuclear‐to‐cytoplasmic ratio was moderate to marked. No mitotic figures or evidence of inflammation were observed. Given the cellular features on cytology, the primary differential diagnoses were either a lipid‐rich mesenchymal (i.e., liposarcoma) or, less likely, lipid‐rich malignant epithelial (i.e., sebaceous gland carcinoma) neoplasm.

Incisional skin biopsies at the cranial, caudal, dorsal and ventral borders of the mass were fixed in 10% neutral buffered formalin and routinely processed for histological evaluation. All four samples exhibited similar histologic findings. The dermis was expanded and effaced by a densely cellular, poorly demarcated and infiltrative neoplastic cell population arranged in solid aggregates or interlacing streams separated by a delicate fibrovascular stroma (Figure 2). The neoplastic cells were polygonal to spindle‐shaped, medium‐sized to large, had variably distinct borders, and contained small to moderate amounts of a finely granular, eosinophilic cytoplasm. Occasional neoplastic cells exhibited either multiple, small, round to oval, variably discrete and clear intracytoplasmic vacuoles or a single, large, oval, discrete, clear intracytoplasmic vacuole that compressed the nuclei toward the periphery. The nuclei were often central and occasionally paracentral, oval, medium‐sized to large, with stippled chromatin and one to two variably prominent nucleoli. Anisocytosis and anisokaryosis were marked, and karyomegaly was moderate. One mitotic figure was observed in a 2.37 mm^2^ area. No evidence of inflammation, haemorrhage or necrosis was observed. Histopathology confirmed the diagnosis of a well‐differentiated, low‐grade subcutaneous liposarcoma in the dog's proximal right thoracic limb.

The animal was referred to the institution's oncology service, and limb amputation was recommended. The owner declined surgical treatment, chemotherapy and radiotherapy and opted for palliative care. Gabapentin (10 mg/kg PO, q 12 h for 30 days) was prescribed for pain management. Two months after the initial visit, the mass was unchanged in size and appearance. A follow‐up CBC revealed severe regenerative anaemia characterized by macrocytosis and reticulocytosis, marked leucocytosis with neutrophilia but no left shift, mild lymphopenia and severe thrombocytopenia (Table 1). Serum chemistry revealed severe hepatocellular injury and marked azotaemia (Table 1). Approximately 1 month later, the owner reported that the dog had spontaneously died at home. Efforts to pursue a postmortem examination were unsuccessful, as the owner declined.

## 3 Discussion

Despite the dog's absence of lameness and overall good health, the rapidly growing subcutaneous mass, clinicopathologic abnormalities and gross appearance of the mass (i.e., firm consistency with a soft, depressed centre) strongly suggested a malignant neoplasm. Differential diagnoses considered included soft tissue sarcoma, rhabdomyosarcoma, haemangiosarcoma, osteosarcoma, chondrosarcoma and lymphoma (Dennis et al. 2011; Hendrick 2016; Avallone et al. 2021). Inflammatory or infectious causes were considered less likely due to the rapid progression, absence of fever and lameness, mild pain on palpation and gross appearance of the mass.

Although mitotic figures were absent on cytology, the high cellularity, lipid‐rich background, cellular morphology, arrangement and degree of atypia supported the clinical suspicion of malignancy, raising concern for a lipid‐rich neoplasm such as liposarcoma or, less likely, sebaceous gland carcinoma. The cellular features on cytology and differential diagnoses considered in this case are consistent with the previous cytological diagnosis of well‐differentiated and pleomorphic liposarcomas (Romsland et al. 2011; McAloney et al. 2020). Histopathology confirmed the diagnosis of a Grade I soft tissue sarcoma, most consistent with a well‐differentiated, low‐grade subcutaneous liposarcoma. Consistent with previous established diagnostic criteria, the diagnosis of a well‐differentiated, low‐grade liposarcoma in this case was based on the cell morphology, predominant cellular arrangement, degree of atypia, low mitotic activity and absence of necrosis (Saik et al. 1987; McCarthy et al. 1996; Baez et al. 2004; Vascellari et al. 2004; Frase et al. 2009; Dennis et al. 2011; Hendrick 2016). Special stains such as Oil Red O and Sudan III, which highlight intracellular lipids, and immunohistochemistry may aid the diagnosis of liposarcomas (Wang et al. 2005; Masserdotti et al. 2006; Frase et al. 2009; Dennis et al. 2011; Diep and Fleis 2012), but these techniques were not performed in this case. Paraffin embedding vastly reduces cellular lipid content, thereby precluding the use of Oil Red O and Sudan III staining. While immunohistochemistry is helpful (Dennis et al. 2011), no validated immunohistochemical panel currently exists for the definitive diagnosis of canine liposarcomas, which limits its utility. Diagnosis, subtyping and grading of canine liposarcomas are typically based on routine histopathology, as in this case (Dennis et al. 2011; Hendrick 2016).

Canine liposarcomas are classified by cellular morphology into well‐differentiated, myxoid and pleomorphic variants, using terminology analogous to that used in human pathology (Weiss and Goldblum 2008; Hendrick 2016). Well‐differentiated liposarcomas in both species are often immunopositive for MDM2 and CDK4, and consistently immunonegative for p53 (Weiss and Goldblum 2008; Avallone et al. 2016; Sciot 2021; Muscatello et al. 2024). In humans, MDM2 immunopositivity is associated with *MDM2* gene amplification and is a diagnostic marker for both well‐differentiated and dedifferentiated subtypes (Weiss and Goldblum 2008; Sciot 2021). In dogs, however, this association is weaker, as MDM2 immunopositivity occurs across liposarcoma subtypes irrespective of *MDM2* gene amplification (Avallone et al. 2016; Muscatello et al. 2024). These findings suggest that, despite morphologic similarities, canine liposarcomas are likely biologically distinct, with unique genetic and proteomic signatures.

In humans, the histologic grade of soft tissue sarcomas is the most reliable predictor of metastasis and the overall 5‐year survival rate (Coindre et al. 2001; Coindre 2006). In contrast, although the histologic grade of soft tissue sarcomas in dogs helps predict recurrence and metastatic risk, its association with survival time is unclear (Dennis et al. 2011). Moreover, evidence of metastasis at or after surgery has not consistently indicated a poor prognosis (Baez et al. 2004; Dennis et al. 2011). In this case, despite no detectable metastases on imaging and diagnosis of a well‐differentiated, low‐grade liposarcoma, metastatic disease was suspected based on the dog's progressive clinical decline, severe haematologic and biochemical abnormalities and spontaneous death approximately three months after diagnosis. Similar to other canine soft tissue sarcomas, particularly low‐grade variants, liposarcomas are generally considered to have low metastatic potential (Baez et al. 2004; Dennis et al. 2011). Nonetheless, metastases to regional or distant lymph nodes, lungs, spleen, liver and kidneys have been documented (Kuntz et al. 1997; Baez et al. 2004; Wang et al. 2005; Frase et al. 2009; Cramer et al. 2011; Diep and Fleis 2012; Gower et al. 2015). Unfortunately, the absence of postmortem examination in this case precludes definitive grading and staging. Whether death resulted from metastatic disease, albeit likely, or unrelated causes remains unknown.

Limb amputation was recommended but declined by the owner, thereby limiting histopathological evaluation to a few biopsy samples. Although accurate grading based on biopsy remains possible, as low‐grade soft tissue sarcomas and well‐differentiated liposarcomas can metastasize (Kuntz et al. 1997; Cramer et al. 2011; Linden et al. 2019), limited sampling in this dog may have resulted in inaccurate tumour grading and incomplete staging, particularly given the dog's clinical decline and outcome. A more comprehensive assessment of the neoplasm, such as that afforded by limb amputation, might have revealed a higher histologic grade, enabled early detection of metastatic disease to regional lymph nodes, and improved prognostic accuracy.

Complete surgical excision is the preferred treatment for liposarcomas, resulting in improved survival compared to marginal or incomplete resection (Baez et al. 2004; Dennis et al. 2011; Hendrick 2016). Although the prognostic significance of histologic subtype in canine soft tissue sarcomas, including liposarcomas, remains unconfirmed, survival time is associated with the completeness of surgical margins (Baez et al. 2004; Hendrick 2016). A previous study demonstrated that dogs undergoing wide excision for liposarcoma had a significantly longer median survival (1188 days; range: 5–1746) compared to those receiving marginal (649 days; range: 1–1782) or incomplete (183 days; range: 2–733) excision (Baez et al. 2004). The approximately 90‐day survival in this case, following only incisional biopsies and palliative care, is consistent with outcomes reported for incomplete excision. Moreover, delaying or refusing treatment may significantly reduce survival, even in tumours that appear well differentiated.

Early diagnosis, accurate tumour grading, complete staging and timely surgical intervention are crucial for optimizing the outcomes of canine soft tissue sarcomas. Although well‐differentiated liposarcomas may appear clinically indolent, their local invasiveness can limit treatment options if referral is delayed. Even low‐grade variants may have systemic effects or undergo malignant progression. Therefore, liposarcoma should be considered as a differential diagnosis in dogs with subcutaneous masses in the proximal limbs. Diagnostic workup should include imaging, cytology, histopathology and prompt surgical referral. Complete excision, potentially requiring amputation, is indicated based on tumour extent.

## Author Contributions

P.B. reviewed the morphologic diagnosis, drafted the manuscript, prepared the table and figures and reviewed the final version of the manuscript. T.R., B.A. and H.F. established the clinical diagnosis and clinical recommendations and reviewed the final version of the manuscript. D.C. established the pathologic diagnosis, prepared the figures and reviewed the final version of the manuscript.

## Funding

The authors have nothing to report.

## Ethics Statement

The authors confirm that the journal's ethical policies, as noted in the journal's author guidelines webpage, have been adhered to. Ethical approval was not required as this was a single case report from our institution. The animal was treated according to our institution's veterinary standard of care and ethics requirements.

## Conflicts of Interest

The authors declare no conflicts of interest.

## Data Availability

No datasets were generated or analysed for this case report.
